# Make or break: the thermodynamic equilibrium of polyphosphate kinase-catalysed reactions

**DOI:** 10.3762/bjoc.18.134

**Published:** 2022-09-20

**Authors:** Michael Keppler, Sandra Moser, Henning J Jessen, Christoph Held, Jennifer N Andexer

**Affiliations:** 1 Institute of Pharmaceutical Sciences, University of Freiburg, Albertstr. 25, 79104 Freiburg, Germany; 2 Institute of Organic Chemistry, University of Freiburg, Albertstr. 21, 79104 Freiburg, Germany; 3 Department of Biochemical and Chemical Engineering, TU Dortmund University, Emil-Figge-Str. 70, 44227 Dortmund, Germany

**Keywords:** ATP regeneration, biocatalyst, ePC-SAFT, polyp, PPK

## Abstract

Polyphosphate kinases (PPKs) have become popular biocatalysts for nucleotide 5'-triphosphate (NTP) synthesis and regeneration. Two unrelated families are described: PPK1 and PPK2. They are structurally unrelated and use different catalytic mechanisms. PPK1 enzymes prefer the usage of adenosine 5'-triphosphate (ATP) for polyphosphate (polyP) synthesis while PPK2 enzymes favour the reverse reaction. With the emerging use of PPK enzymes in biosynthesis, a deeper understanding of the enzymes and their thermodynamic reaction course is of need, especially in comparison to other kinases. Here, we tested four PPKs from different organisms under the same conditions without any coupling reactions. In comparison to other kinases using phosphate donors with comparably higher phosphate transfer potentials that are characterised by reaction yields close to full conversion, the PPK-catalysed reaction reaches an equilibrium in which about 30% ADP is left. These results were obtained for PPK1 and PPK2 enzymes, and are supported by theoretical data on the basic reaction. At high concentrations of substrate, the different kinetic preferences of PPK1 and PPK2 can be observed. The implications of these results for the application of PPKs in chemical synthesis and as enzymes for ATP regeneration systems are discussed.

## Introduction

**Figure 1:**
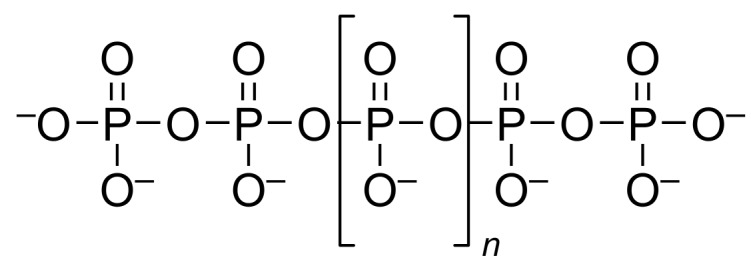
Polyphosphate, a ubiquitous phosphate storage molecule. Reported chain lengths range from three to several thousands.

**Figure 2:**
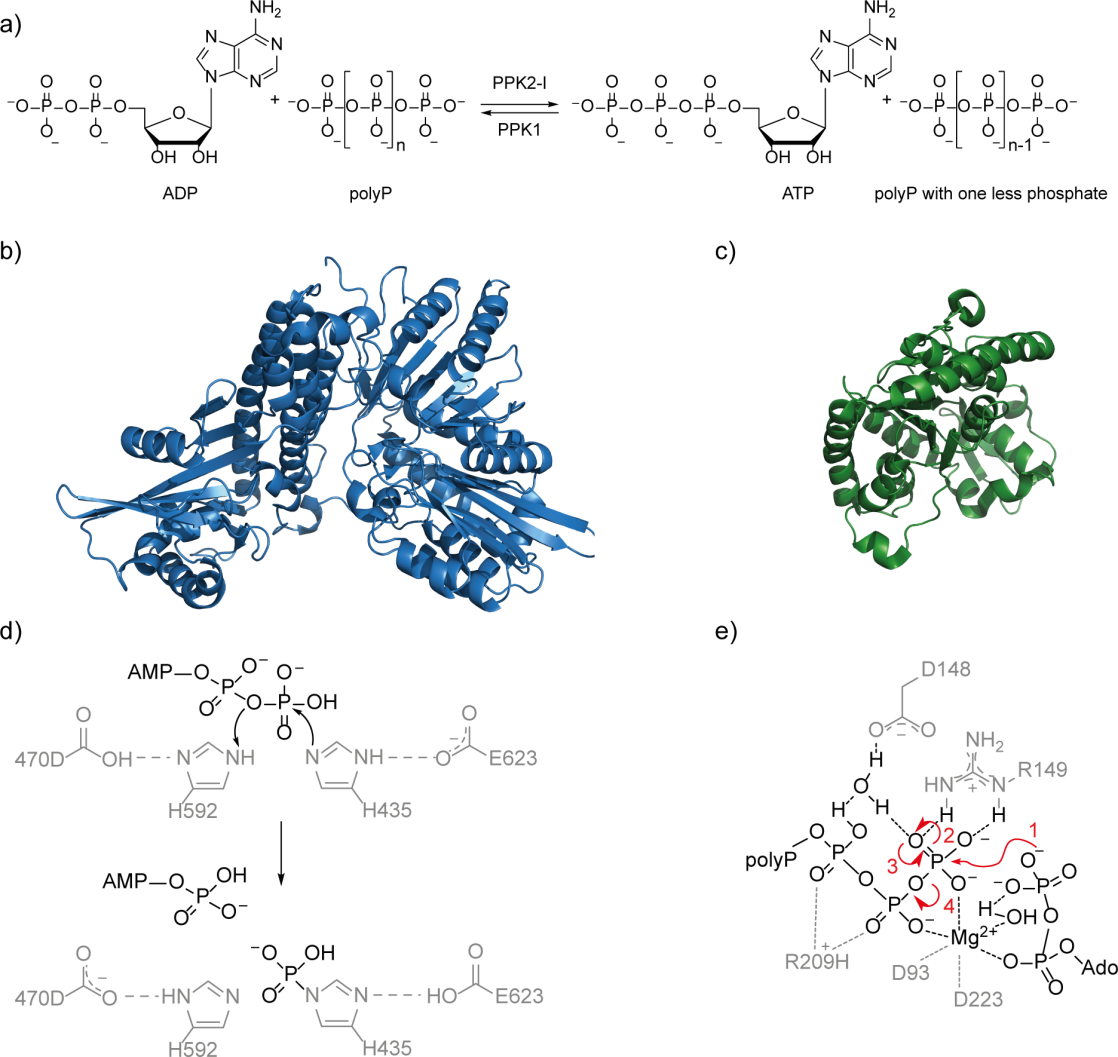
Comparison of PPK1 and PPK2 enzymes. a) Reaction scheme; b) structure of the *Ec*PPK1 monomer (PDB 1XDO) [13]; c) structure of the *Sm*PPK2 monomer (PDB 3CZQ) [10]; d) proposed mechanism of PPK1 with phosphate transfer via a phosphor enzyme intermediate; e) PPK2 mechanism exemplarily shown for the associative reaction pathway.

Polyphosphate (polyP, Figure 1) is a linear polymer of up to thousands of phosphate residues connected by phosphate anhydride bonds. It serves as a phosphate storage molecule and plays a crucial role in biofilm formation and stress responses of cells [1]. So far polyP has been detected in every living organism investigated [1–3]. In 1956, Kornberg described the first polyP kinase (PPK) in *Escherichia coli* catalysing adenosine 5’-triphosphate (ATP)-dependent synthesis of polyP (Figure 2a) [4]. The enzyme was reclassified as family-1 PPK (PPK1) when a structurally different PPK (family-2, PPK2) was found in *Pseudomonas aeruginosa* in 2002 [5]. PPK2 were later subdivided into three classes: PPK2-I, PPK2-II, and PPK2-III phosphorylating nucleotide diphosphates (NDPs), nucleotide monophosphates (NMPs), and both, respectively [6]. Nevertheless, these substrate profiles rather seem to be preferences, as most enzymes catalyse all phosphorylation steps during extended reaction times; also higher phosphorylated species have been detected in the reactions [7–8]. The enzymes characterised from *E. coli* (*Ec*PPK1) and *Sinorhizobium meliloti* (renamed *Ensifer meliloti, Sm*PPK2) are often regarded as model enzymes for PPK1 and PPK2 [9–10]. From a structure perspective, PPK1 enzymes form tetramers in solution with a mass of approximately 80 kDa for the monomer (Figure 2b). Although not being an integral membrane protein, the enzyme is described to be membrane-associated [11–13]. The phosphate transfer likely proceeds via formation of a phospho-enzyme intermediate (Figure 2d). Two essential histidine residues for autophosphorylation were identified by mutagenesis experiments [9,13–14]. Variants carrying mutations at these histidine residues lost the ability to synthesise polyP or ATP in vitro*,* clearly demonstrating the necessity of the residues for catalysis [14]. PPK2-I enzymes are of lower molecular weight than their PPK1 counterparts, with an approximate molecular mass of 40 kDa for a monomer (Figure 2c) [5]. They form dimers or tetramers in solution and are not purified from membrane fractions [5,10,15–16]. Based on the crystal structures of three PPK2-III, the coordination of polyP and ADP by positively charged amino acids (lysine and arginine) has been suggested [16–17]. Two magnesium ions are held in place by two conserved aspartate residues that further coordinate the polyP and ADP for an in-line reaction of these two substrates. Out of this arrangement two reaction pathways have been discussed, an associative and a dissociative one. The associative one is an S_N_2-like attack of ADP on the terminal phosphate of the polyP chain, while the dissociative one is an S_N_1-like reaction where the terminal phosphate dissociates from the polyP chain before being attacked by the nucleotide [18]. Both mechanisms could proceed without a phosphate group transfer onto an amino acid side chain of the enzyme as in PPK1: here, the enzyme structure generates proximity and polarisation of substrate bonds (Figure 2e) [17]. Apart from the structures, the kinetic preference of either polyP synthesis (NTP usage) or NTP synthesis (polyP degradation) has been described to be a characteristic feature of PPK1 and PPK2(-I), respectively (Figure 2a). This is supported by analysis of the kinetic parameters *K*_M_ and *v*_max_ of selected enzymes (Table S6, Supporting Information File 1) [5,10–11,15,19–21]. A sequence-based classification of PPKs is in most cases straightforward and unambiguous. Nevertheless, there seem to be exceptions: regarding the amino acid sequence, the PPK1 from *Vibrio cholerae* is very similar to the one from *E. coli* with 82% similarity (64% identity) on the amino acid level; however, it was described to show kinetic preferences of a PPK2 [20]. While the PPK2 from *P. aeruginosa* catalyses both synthesis and usage of ATP with kinetic preference for ATP synthesis, the “model PPK2” from *S. meliloti* (*Sm*PPK2) only tested positively for ATP synthesis [5,10]. The PPK2 from *Corynebacterium glutamicum* (*Cg*PPK2) has kinetic preferences of a PPK1 although being a PPK2 regarding the amino acid sequence [15].

**Figure 3:**
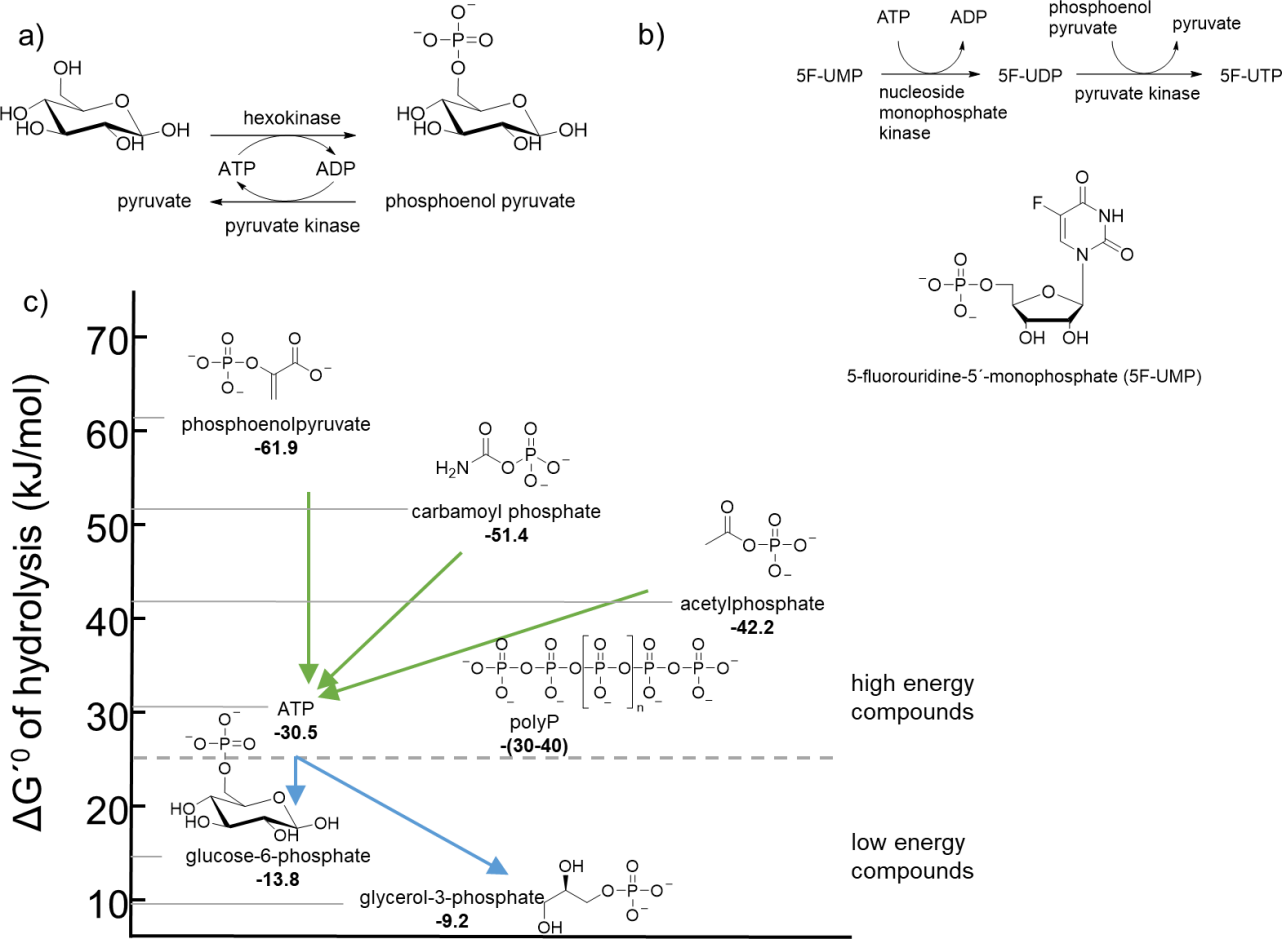
a) Kinases as ATP regeneration enzymes, exemplified by the hexokinase-catalysed ATP-dependent phosphorylation of glucose. The ADP formed is converted back to ATP by pyruvate kinase using phosphoenol pyruvate as the phosphate donor [37]. b) Utilisation of kinases for biocatalytic NTP production. Usually, a sequence of different kinases with a suitable phosphate donor is used, depending on the starting material different enzymes have to be employed. The example shows the final two reaction steps in the biosynthesis of 5-fluorouridine-5`-monophosphate (5F-UMP). The phosphorylation of the NMP is catalysed by the ATP dependent nucleoside monophosphate kinase yielding a 5F-UDP and ADP. 5F-UDP is then phosphorylated by pyruvate kinase under consumption of phosphoenol pyruvate [42]. c) Ranking of different phosphate donors that can be used for ATP regeneration or production because their phosphate group transfer potential is higher compared to ATP. From ATP the phosphate group can be transferred onto various substrates but the phosphate group cannot be easily transferred back to ADP from a low energy compound [28–30].

The biocatalytic activity of PPKs can be used as a tool for the regeneration of ATP (and other NTPs, Figure 3a) as well as for the biocatalytic production of modified NTPs (Figure 3b) [22–23]. Compared to other ATP regeneration systems using phosphate donors such as phosphoenolpyruvate, carbamoyl phosphate and acetyl phosphate, PPK catalysed reactions benefit from their stable and inexpensive phosphate donor polyP [24]. Besides the difference among PPK families, further process parameters determine the kinetic preference towards ATP synthesis or utilisation. Especially for synthetic reactions with the aim to produce and isolate phosphorylated product, the initial substrate/product ratio is an important parameter for kinetics and for the reaction equilibrium, as it defines how much conversion will be achieved [25]. For acetate kinase a conversion of at least 90% using stoichiometric amounts of ADP and acetylphosphate was reported [26]. The reaction of pyruvate kinase (phosphoenolpyruvate as phosphate donor) is strongly favouring ATP synthesis both in vivo and in vitro, this reaction was originally considered to be irreversible in cells and a point of flux control. Newer findings showed the reaction to be actually an equilibrium, although positioned far on the product side [27–29]. Also for carbamate kinase (using carbamoyl phosphate), the equilibrium lies far on the ATP side with a calculated equilibrium concentration of 3.9 × 10^−4^ M ADP out of 0.1 M ADP [30]. As most kinases use a phosphate donor with a high phosphate transfer potential (see Figure 3c) and a product which has to be lower in its phosphate transfer potential, the overall outcome of the reactions is expected [28–29,31–32]. The phosphate transfer potential is a measure of tendency of a molecule to transfer a phosphate group onto an acceptor molecule. A high phosphate transfer potential refers to a high energy release when the phosphate group is hydrolysed. With PPKs, this seems slightly different: The hydrolysis energy involving the terminal phosphate group of polyP (Δ^R^*G*^0^ ≈ −30 kJ/mol) and internal phosphate groups (Δ^R^*G*^0^ ≈ −30 to −40 kJ/mol) is comparable to the standard hydrolysis energy of ATP [33–34]. Under physiological conditions as well as in an in vitro system the actual Δ*G* may be different by coordination of cations and the ionic strength, temperature and pH of the reaction solution [29,35–36]. Compared to other ATP synthesis reactions very little is known about the thermodynamics for the PPK1 and PPK2-catalysed reactions with polyP as a phosphate donor since studies mainly focus on the kinetic characterisation of these enzymes.

Considering the growing interest in the application of PPK enzymes, knowledge about the thermodynamic course of the reaction would be useful for the optimisation of biocatalytic syntheses of nucleotides as well as nucleotide regeneration systems (Figure 3a and 3b). In a regeneration system (exemplarily shown for the hexokinase-catalysed phosphorylation of glucose (Figure 3a) the formed ADP has to be converted back to ATP to maintain a sufficient pool of ATP for the hexokinase reaction [37]. Each of the phosphate donors discussed can be used in combination with the corresponding kinase to regenerate the ATP rendering the available regeneration systems flexible and broadly applicable. While the depicted regeneration system is quite simple, the reaction can be embedded in complex biosynthetic networks such as in vitro *S*-adenosylmethionine (SAM)- or carbon dioxide fixation cycles, and de novo nucleobase synthesis [38–41]. For the biocatalytic synthesis of ATP or derivatives, up to three consecutive phosphorylation reactions are coupled in a linear cascade to produce the desired NTP. Figure 3b shows the reaction sequence from 5-fluorouridine-5’-monophosphate to the triphosphate in an enzymatic synthesis of an unnatural uridine nucleotide [42]. In these type of setup, the yield of the overall reaction is strongly determined by the position of the thermodynamic equilibrium of the last reaction step [22–23,42–45]. To efficiently implement such a reaction sequence, detailed kinetic and thermodynamic information has to be available to identify bottlenecks and improve the turnover of such cascade systems [25].

In the present study, we analysed a set of well-known PPK enzymes regarding the thermodynamic equilibrium of ATP synthesis and compared the experimental results obtained with theoretical calculations. The theoretical calculations addressed the equilibrium position of the considered reactions as function of the substrate concentration. In addition to the general question of the equilibria of the PPK-catalysed reactions in comparison to other kinases, we sought to evaluate the contribution of the reported characteristic “preferences” of PPK1 and PPK2 enzymes regarding polyP synthesis and polyP degradation on the reaction rate and equilibrium formation.

## Results and Discussion

The enzymes investigated include all four kinds of identified PPK1/PPK2 enzymes. PPK1 from *E. coli* (*Ec*PPK1) and *V. cholerae* (*Vc*PPK1) [20] are two well investigated PPK1 with different kinetic preferences [20–21]. *Sm*PPK2 [10] is the model PPK2 enzyme and often described as the counterpart of *Ec*PPK1. *Cg*PPK2 is also designated to have different kinetic preferences compared to the model enzyme *Sm*PPK2, favouring the usage of polyP over the synthesis of ATP [15]. All enzymes used in this work were produced in *E. coli*. While the PPK2s were produced as soluble enzymes and could be easily purified via Ni-NTA affinity chromatography using N-terminal His-tags, the PPK1 enzymes required an N-terminal maltose binding protein (MBP-tag) to improve solubility [46–47]. Trials to cleave the MBP-tag were unsuccessful and resulted in inactive protein aggregates. It has been shown that large tags such as the MBP-tag, as well as the positioning (N- or C-terminal) of the tag can influence the activity of an enzyme [48]; nevertheless, its thermodynamic characteristics should not be affected. Consequently, we decided to use the enzymes with the tags, as this likely is the most pragmatic and straightforward preparation of the enzymes for their use in chemical synthesis. The assay setup used for all PPKs is similar to established ATP regeneration systems with PPK2 enzymes, with a 10:1 excess of polyP (calculated as single phosphates [49]) over the nucleotide (default concentration in this work 2 mM) [39,50–52]. This is also a realistic scenario for biosynthetic reactions using PPKs for the production of NTPs [22,52]. The excess of polyP should prevent depletion of sufficiently long polyP chains since acceptance of very short chains (*n* < 10) might differ between different PPKs [10,15].

**Figure 4:**
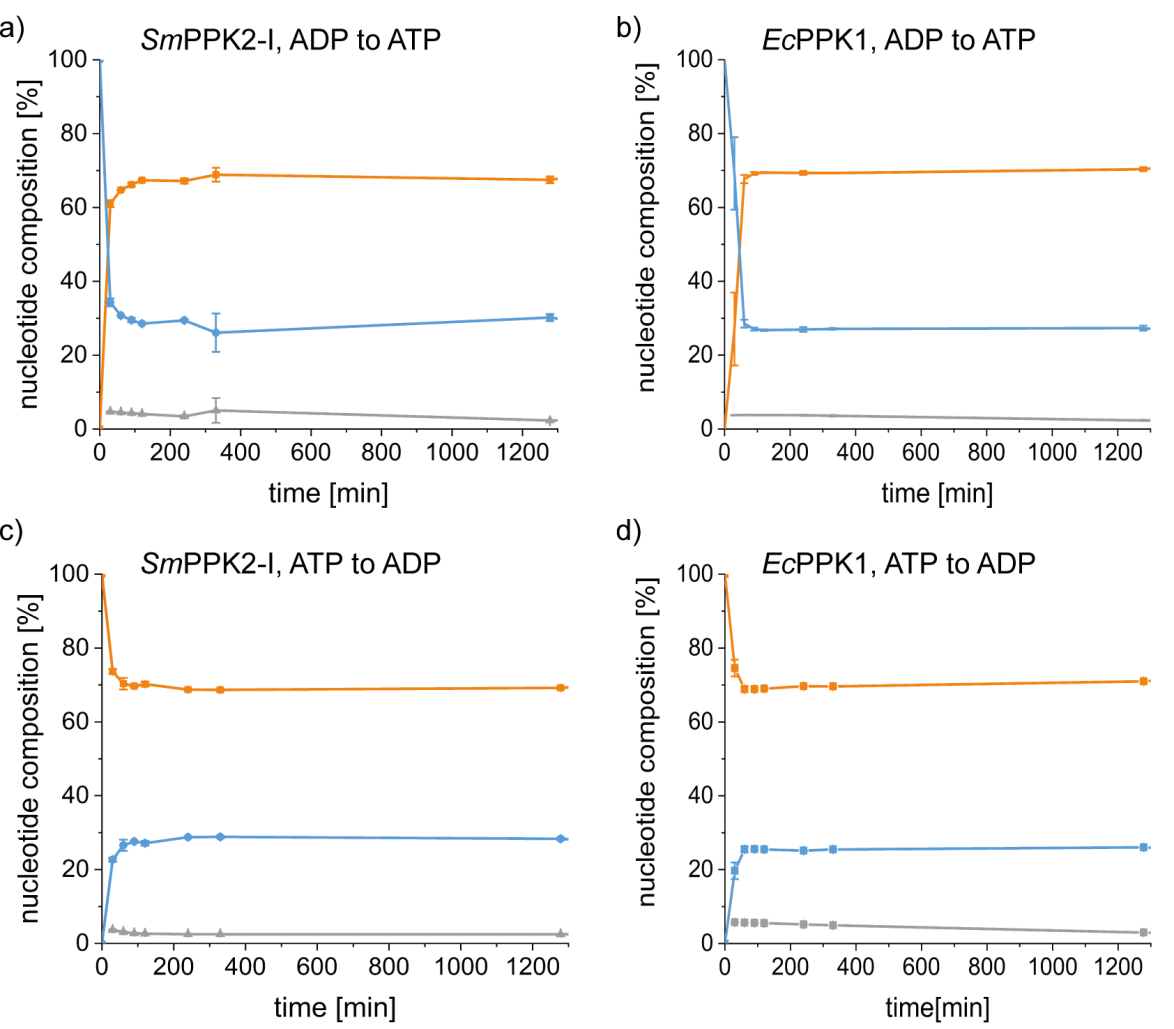
Time courses of reactions started with ADP catalysed by a) *Sm*PPK2 and b) *Ec*PPK1. Time courses of reactions started with ATP catalysed by c) *Sm*PPK2 and d) *Ec*PPK1. The nucleotide concentration was 2 mM, the reaction was carried out at pH 8.0 and 37 °C. Grey = AMP, blue = ADP, orange = ATP; the nucleotide composition is given in mol %.

First experiments were conducted with the “model PPKs” *Ec*PPK1 and *Sm*PPK2. At 37 °C and pH 8, each enzyme was incubated with either ADP or ATP and polyP as co-substrate; the resulting nucleotide distributions were analysed by HPLC as a function of time (Figure 4). In all experiments, the equilibrium was reached after 90 minutes with no further changes in product concentrations upon extending incubation time. Regardless of starting the reaction with ADP or ATP, the equilibrium tends towards a ratio of 70% ATP and 30% ADP (molar ratio, ADP/ATP = 0.43). About 5% AMP was observed during the reaction, which is derived from the starting material and is not further accumulating over the course of the reaction. Higher phosphorylated compounds such as adenosine 5'-tetraphosphate, which are additional reaction products of PPK2 catalysed reactions, were not detected under the conditions applied, this usually requires higher enzyme concentrations [7–8]. A similar equilibrium concentration was observed for *Vc*PPK1 (ADP/ATP 30%:70%, Figure S1, Supporting Information File 1). In the *Cg*PPK2-catalysed reaction, the ADP/ATP ratio (ADP:ATP 35–40%:65–60%, Figure S1, Supporting Information File 1), as well as the amount of AMP formed was slightly higher compared to the other PPKs, especially when starting from ADP as substrate. This suggests that *Cg*PPK2 possesses a pronounced myokinase (2 ADP ↔ ATP + AMP) activity that cannot be suppressed at the applied conditions nor separated from the main reaction; this has been already described as a side reaction for other PPK2s [7,17]. In summary, these findings demonstrate that both, PPK1 and PPK2 enzymes catalyse the formation of the same equilibrium despite their different reaction pathways. After 30 minutes the reaction was close to equilibrium formation; thus, no kinetic effect of family 1 or 2 could be observed. The results obtained also show that polyP, despite its rather low phosphate transfer potential, is a sufficient phosphate donor for the phosphorylation of ADP, as the thermodynamic equilibrium is clearly positioned on the ATP side.

Next, we analysed the influence of the amount of the starting nucleotide for the two model enzymes *Ec*PPK1 and *Sm*PPK2: either one quarter (0.5 mM) or twice (4 mM) the default amount (2 mM) of nucleotide was used. With 0.5 mM, the equilibrium composition contained slightly lower amounts of ATP than with 2 mM after 30 min (60% ATP, Figure 4b, Figure S2, Supporting Information File 1). With 4 mM of starting nucleotide, the equilibrium is composed similar to the one of the experiments with 2 mM nucleotide (70% ATP/30% ADP, Figure S3, Supporting Information File 1). This can be explained by a concentration effect of the nucleotide on the reaction equilibrium. For this, we applied the thermodynamic activity-based framework that uses the equilibrium constant *K*_a_, which is independent of concentration. It is expressed via the law of mass action, and exemplarily for the reaction from ADP to ATP it reads as


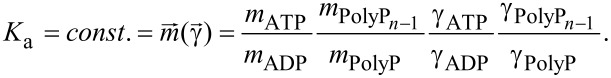


**Figure 5:**
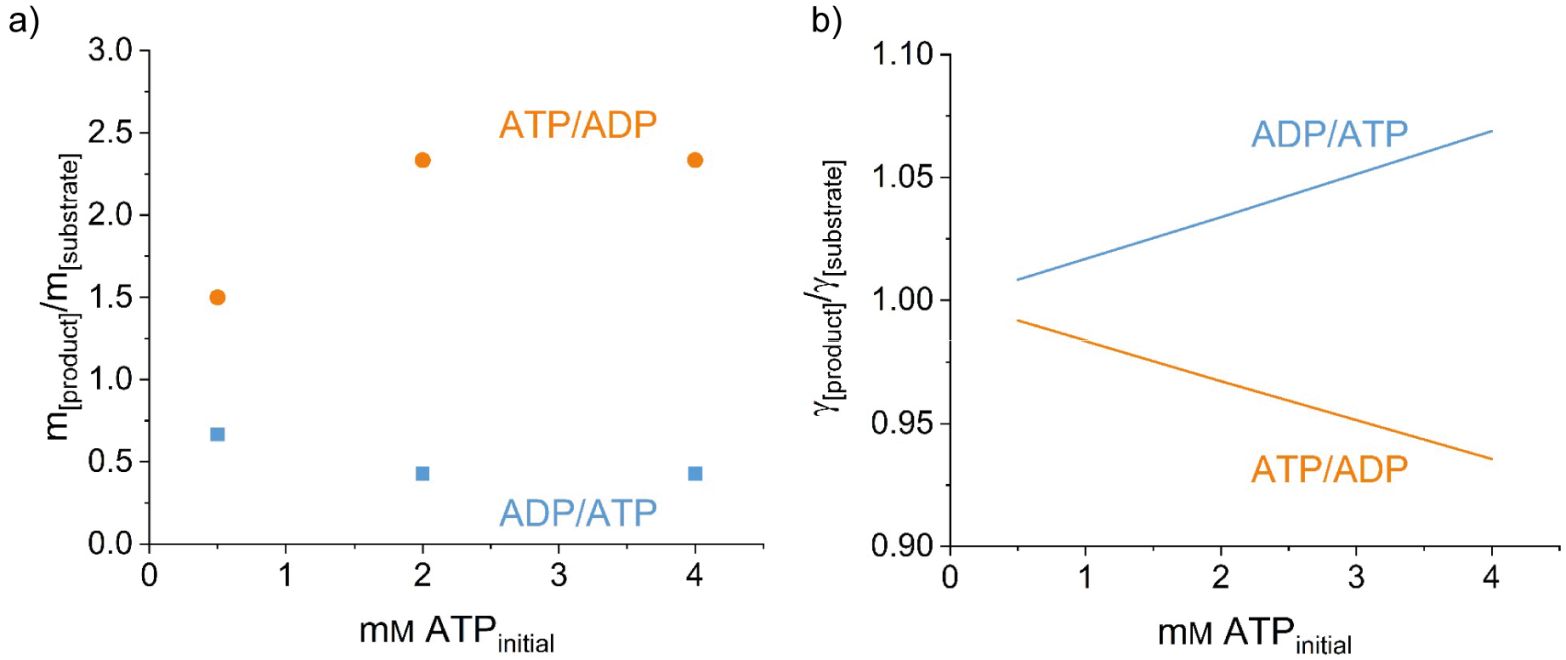
a) Ratio of product to substrate for the three nucleotide concentrations (0.5, 2, and 4 mM) used in this work, both synthesis directions are depicted (orange: ATP as substrate, blue: ADP as substrate). a) The ratio (moles per kg) is shown against the concentration of initial ATP. b) ePC-SAFT predicted ratio of activity coefficients for the two reactions against the concentration of initial ATP.

This equation shows that any change in the reaction equilibrium (ratio of the equilibrium molalities 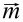) must be equalised by the ratio of the equilibrium activity coefficients 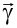 of the reacting agents, or in other words 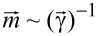. The activity coefficients describe the molecular interactions among the reaction participants in the reaction mixtures, which has been established for biochemical reactions [53–54]. The predictive electrolyte equation of state ePC-SAFT [55] was applied to predict the activity coefficients at equilibrium. ePC-SAFT is an electrolyte perturbation theory which describes physical interactions by accounting for molecular repulsion and attraction caused by van-der-Waals forces, hydrogen bonding, and Coulomb forces. The ePC-SAFT parameters of the nucleotides were fitted in previous works to experimental osmotic pressures of pseudo-binary mixtures of nucleotide and water [29,33]. As modelling polyP with high chain length is currently not possible with ePC-SAFT, we assumed that only nucleotides were present in water. The consequence of this assumption is that interactions among nucleotides and polyP were considered to be equal to interactions among nucleotides and polyP*_n_*_−1_, and we focused only on the ratio of the nucleotides. Upon increasing the nucleotide concentration, the equilibrium concentration ratios shift to the ATP side of the reactions: the higher the initial substrate concentration the lower the equilibrium ratio ADP/ATP that is to be expected. This fits with the experimental data shown in Figure 5a (experimental concentration ratio at equilibrium over the initial nucleotide concentration). It can be observed from Figure 5b that the ePC-SAFT predictions are in qualitative agreement with the experimental findings, since the activity-coefficient ratio behaves reciprocally to the experimentally observed concentration ratio at equilibrium (Figure 5a). Thus, the interactions between the reacting agents (covered by 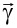) cause a shift in the equilibrium position 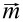 according to the results shown in Figure 5a fulfilling the above-shown thermodynamic constraint 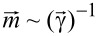. As mentioned before, the theoretical prediction procedure represented in Figure 5b ignores the presence of polyP in the reaction mixture. However, in the reaction also polyP*_n_* and polyP*_n_*_−1_ take part; their concentration ratio might additionally influence the equilibrium position of the overall reaction. As it is not yet possible to characterise polyP by thermodynamic modelling due to lack of experimental data and knowledge of the precise distribution of chain lengths, in a second step the influence of orthophosphate as a representative for polyP was investigated on the qualitative behaviour of the results in Figure 5b. The results are not shown in detail here, but we found that the addition of orthophosphate did not change the qualitative course of the activity-coefficient ratios from Figure 5b.

Despite the qualitative success of the ePC-SAFT predictions, some quantitative discrepancies can be observed between Figure 5a and 5b. Using 4 mM of substrate or above, no further change in the ADP/ATP equilibrium was experimentally observed (Figure 5a), while ePC-SAFT predicts a linear behaviour with nucleotide concentration (Figure 5b). This discrepancy between model and experiment might be explained by experimental issues (measurement uncertainty, occurrence of side reactions not considered in the modelling) or by theory issues, since, as described before, the influence of polyP was neglected in modelling with ePC-SAFT. Further, it should be noted again ePC-SAFT was used in a predictive mode, which was not fitted at all to any reaction experiment.

**Figure 6:**
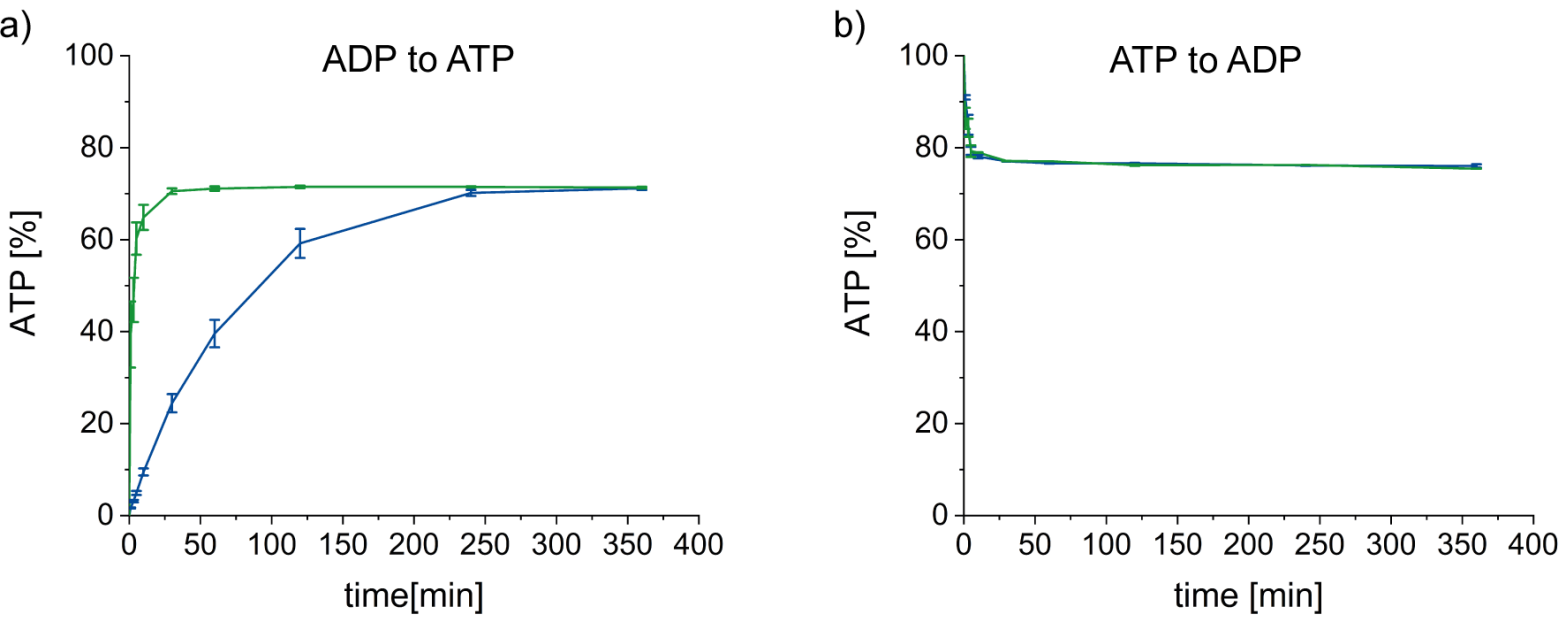
Time courses comparing *Ec*PPK1 (blue) and *Sm*PPK2 (green) for ATP synthesis (a) and ATP degradation (b) when 4 mM of the nucleotide was provided. While there is very little difference in the degradation reaction, *Sm*PPK2 reaches the equilibrium earlier than *Ec*PPK1 in the synthesis reaction.

In contrast to the reaction equilibria, substantial kinetic differences between PPK1 and PPK2 were observed at 4 mM substrate concentration regarding the ATP synthesis reaction. Starting from ADP, the *Sm*PPK2-catalysed reaction reached the equilibrium in 30 minutes, the same reaction catalysed by *Ec*PPK1 only after 240 minutes (Figure 6). Based on literature data (Table S6, Supporting Information File 1), an opposite trend was expected for the reaction started with ATP; however, in this direction, no clear kinetic effect could be observed, and the equilibrium is generally reached very quickly. This effect is experimentally observed – it is shown in the literature that kinetics should be expressed based on the thermodynamic activity of the enzyme [56]. The enzyme activity coefficients were not taken into account in the present work, which does not allow drawing conclusions from the concentration-based kinetics presented in Figure 6 and the activity-based consideration of the equilibrium constants yielding the results in Figure 5. Nevertheless, the experimentally observed difference in velocity agrees with the described kinetic “preference” of PPK2 for nucleotide synthesis and the tendency to use these enzymes for ATP (re)generation systems.

Another variable discussed in the context of PPK reactions is the polyP chain length. Lowered or increased activity with different chain lengths of polyP is described for various PPKs [5,15,19]. So far we used polyP*_n_* with an average chain length of *n* = 20 (as estimated by ^31^P NMR, Figure S5, Supporting Information File 1) for all reactions, therefore the question remained whether or not the polyP chain length has a substantial influence on the thermodynamic equilibrium. For this reason, we conducted a reaction with a commercial polyP_100_ (calculated as single phosphates, average length confirmed by ^31^P NMR, Figure S6, Supporting Information File 1). Compared to the data obtained for the identical reaction setup with the polyP_20_ (Figure 2), no differences were observed (Figure S4, Supporting Information File 1).

As discussed before, the conversion of a nucleotide diphosphate to the corresponding triphosphate is normally the last step in the biomimetic synthesis of NTPs, and might have a substantial influence on the yield of the whole cascade. A biomimetic cascade published by Whitesides and co-workers for the synthesis of ATP from adenosine uses an acetate kinase for the final conversion of ADP to ATP (Table 1) [45]. Acetylphosphate has long been preferred as phosphate donor over phosphoenolpyruvate in large-scale reactions, due to economic factors such as ease of production and atom economy [57]. On an analytical scale, this reaction reached conversions of up to 94%, which agrees with the thermodynamic equilibrium for acetate kinase of at least 90%:10% ATP:ADP [26].

**Table 1:** Comparison of selected enzymatic syntheses of ATP (derivatives). The triphosphate-forming step is in bold, stoichiometrically added substrates are underlined.

	Reaction sequence	Yield of ATP	Reference

(1)	(a) adenosine + ATP → AMP + ADP	94%	R. L. Baughn et al. [45]
	(b) AMP + ATP → 2 ADP		
	**(c) ADP + ** **acetylphosphate** ** → ATP + acetate**		
(2)	(a) adenosine + ATP → AMP + ADP	70–75%	C. Sun et al. [44]
	(b) AMP + polyP*_n_* → ADP + polyP*_n_*_–1_		
	**(c) ADP + ** **polyP** *** _n_ *** ** → ATP + polyP** ***_n_*** **_–1_**		
(3)	(a) 2-Cl-adenine + PRPP → 2-Cl-AMP +PP_i_	80%	J. Frisch et al. [22]
	(b) 2-Cl-AMP+ polyP*_n_* → 2-Cl-ADP + polyP*_n_*_–1_		
	**(c) 2-Cl-ADP+ ** **polyP** *** _n_ *** ** → 2-Cl-ATP + polyP** ***_n_*** **_−1_**		
	(d) 2-Cl-ATP → 2-Cl-dATP		

A comparable one-pot synthesis was recently published by the group of Li (Table 1) using two PPKs (PPK2-II and PPK2-I) with polyP as a phosphate donor for AMP and ADP phosphorylation; this reached yields up to 75% ATP thus supporting the thermodynamic equilibrium for PPKs reported in this study [44]. Interestingly, we obtained similar results when investigating the class-III PPK2 from *Meiothermus ruber* for its polyP-dependent reaction with AMP (via ADP) to ATP (2.5% AMP/27.5% ADP/70% ATP) [16].

In theory, the addition of another, ideally irreversible reaction can be used to pull the equilibrium further to the product side. Another recent application of PPKs in the cascade synthesis of cladribine triphosphate (2-chloro-2'-deoxyadenosine 5'-triphosphate) could be discussed as an example for this (Table 1). Here, PPK2 enzymes are used to produce 2-Cl-ATP which is then reduced to the desired product. The final reduction could be considered as a pull effect on the PPK2 reaction, since it removes 2-Cl-ATP from the PPK2 reaction equilibrium. In fact, the authors observed an (compared to the adenosine-to-ATP cascades) increased yield of 80% cladribine triphosphate, with 2-Cl-dADP being the major byproduct [22]. The incomplete conversion (despite the irreversible reduction step) has been explained by the previously mentioned myokinase side reactivity of PPK2 enzymes [22]. An alternative explanation might be the PPK equilibrium: most PPKs accept a broad range of nucleoside substrates, therefore some 2-Cl-dATP produced could be a substrate for a PPK2 catalysed 2-Cl-dATP/2-Cl-dADP equilibrium.

## Conclusion

PPK2, and also PPK1 enzymes are frequently used in ATP regeneration systems as well as in cascade reactions for NTP biosynthesis [22–23,39,44,50,58–60]. Therefore, the results presented here can serve to further tune and improve these multi-enzyme reactions and the yield of biomimetic NTP synthesis systems. Usually, reactions for polyP-dependent ATP regeneration as well as NTP biosynthesis are carried out under conditions comparable to the ones used for this study [22,39,50,52]. Overall, we can clearly see a preference of the thermodynamic system towards ATP. This behaviour was not impacted by the choice of PPK, regardless of the different reaction mechanisms. The thermodynamic equilibrium of the PPK reaction is not as close to the product side as in other ATP regenerating systems, which corresponds to the phosphate transfer potential of the different phosphate donors. This raises the question if PPK/polyP based systems are actually the best choice for biocatalytic syntheses of NTPs and other phosphorylated compounds, or if a phosphate donor with a higher phosphate transfer potential would be more useful. In our opinion, this has to be tailored to each individual system and depends on the characteristics of the starting material, and factors such as the necessity to purify the end product. Also, especially PPK2s are described to be very flexible regarding the nucleobase, which is not the case for all other kinases [10,17,61]; depending on the system this might compensate for the not ideal conversion rates. Nevertheless, as it becomes evident from the cladribine system, exactly this broad substrate range might be disadvantageous, thus highlighting the necessity to tailor the choice of enzymes to the substrates and reaction sequence in question. In ATP regeneration systems, the equilibrium issue might be less relevant, as the ATP produced will be directly used by the main reaction.

In future, it will be interesting to investigate the detailed reaction mechanism including the effects of the polyP chain length and counter ions as well as to study the thermodynamic activity of the enzymes. Especially in reaction setups where the synthesised ATP is not directly removed by follow up reactions, options to tune the reaction conditions in order to increase the conversion and yields of the final cascade product will be an important aim.

## Supporting Information

File 1 Details of materials and methods and additional figures and tables.
