# Supplementary material for: Make or break: the thermodynamic equilibrium of polyphosphate kinase-catalysed reactions
Source: Beilstein J Org Chem. 2022 Sep 20;18:1278–88. doi: 10.3762/bjoc.18.134 (PMC9520863; doi:10.3762/bjoc.18.134)
Supplement: File 1 — Details of materials and methods and additional figures and tables. [file Beilstein_J_Org_Chem-18-1278-s001.pdf]

BEILSTEIN JOURNAL OF ORGANIC CHEMISTRY

Supporting Information

for

Make or break: the thermodynamic equilibrium of polyphosphate kinase-catalysed reactions

Michael Keppler, Sandra Moser, Henning J. Jessen, Christoph Held and Jennifer N. Andexer

Beilstein J. Org. Chem. **2022,** *18,* 1278–1288. doi:10.3762/bjoc.18.134

Details of materials and methods and additional figures and tables

License and Terms: This is a supporting information file under the terms of the Creative Commons Attribution License (https://creativecommons.org/licenses/by/4.0). Please note that the reuse, redistribution and reproduction in particular requires that the author(s) and source are credited and that individual graphics may be subject to special legal provisions.
The license is subject to the *Beilstein Journal of Organic Chemistry* terms and conditions: (https://www.beilstein-journals.org/bjoc/terms)

Details of Material & Methods

Chemicals:

All chemicals were purchased from either Carl Roth (Karlsruhe, Germany), Acros Organics (Fisher Scientific GmbH, Schwerte, Germany) or Merck (Taufkirchen, Germany). ATP was purchased from Merck (microbial, ≥99%), as well as ADP (microbial, ≥95%). HPLC analysis of ATP and ADP confirmed that the respective nucleotide was the predominant one with over 96% of the absorbance accounting for the desired nucleotide. Impurities detected were nucleotides with a lower phosphorylation grade, so ADP and AMP were detected in ATP, as well as AMP in the purchased ADP. $PolyP_{20}$ was purchased from Merck (Fisher Scientific GmbH, Schwerte, Germany), $polyP_{100}$ from Kerafast (Boston, Massachusetts, USA). Ni-NTA Agarose slurry was purchased from Invitrogen (Fisher Scientific GmbH, Schwerte, Germany).

Cloning:

The expression plasmid for *Ec*PPK1 (pETM41-EcPPK, Addgene plasmid # 38334) was a gift from Dr Florian Freimoser (University of Zurich). The *Sm*PPK2 construct was used as described previously [1]. *Vc*PPK1 and *Cg*PPK2 were purchased as synthetic gene constructs with codon optimisation for *E. coli.* The gene encoding *Cg*PPK2 was cloned in pET-28a(+) vector utilising the NdeI and XhoI restriction sites of the vector (Table S1). *Vc*PPK1 was cloned in pMAL-c5X using the In-Fusion system. Both gene constructs were amplified by PCR with Phusion High-Fidelity DNA Polymerase. The gene for *Cg*PPK2 was amplified with primers having overhangs with the appropriate restriction sites. *Vc*PPK2 was amplified with a 15 base pair overhang which is complementary to the linearised vector for the following In-Fusion reaction which was conducted according to the manufacturer manual. Successful cloning reactions were confirmed by sequencing (GATC Biotech AG, Ebersberg).

Table S1. List of oligonucleotides used for cloning. In bold, the restriction site is highlighted, underlined regions are complementary to the pMAL vector sequence for In-Fusion cloning.

Oligonucleotide	Sequence (bold=restriction site, underlined=complementary to pMAL)
CgPPK2_NdeI_fwd	TATATATA**CATATG***GTGGGCAAACTG*
CgPPK2_XhoI_rv	TATATATA**CTCGAG***TTAATCACCAATCTG*
VcPPK1_If_fwd	GGGATCGAGGGAAGGATGAGCGCAGATAAACTGTACATCG
VcPPK1_If_rev	TTATTTGAAGCTTATTTACTGGCTGTTATCATTGG
pMAL_fwd	ATAAGCTTCAAATAAAACGAAAGGC
pMAL_rev	CCTTCCCTCGATCCCGAGG

Expression:

Proteins were produced in *E. coli* BL21 Gold(DE3). LB medium (10 mL) containing the respective antibiotic (kanamycin for production of *Ec*PPK1, *Sm*PPK2-I and *Cg*PPK2-I, ampicillin for production of *Vc*PPK1, 50 µg/mL) was inoculated with a single colony from an LB-agar plate. The culture was incubated overnight at 37 °C, 180 rpm in a rotary shaker. This culture was used as a preculture to inoculate fresh LB medium (400 mL) containing antibiotic (50 µg/mL). The preculture was added in a 1% ratio. The culture was incubated at 37 °C, 180 rpm in a rotary shaker. At an optical density (OD_{600}) of 0.5–0.6, gene expression was

induced by addition of IPTG (0.1 mM final concentration). At this point, temperature was lowered to 20 °C and the cells were further cultured for 20 hours. Cells were harvested by centrifugation (Avanti J-26S XP Centrifuge Beckman Coulter, JLA-10.500 rotor, 9000 rpm, 4 °C, 20 min) and stored at −20 °C until further use.

Purification:

For protein purification, cells were resuspended in lysis buffer (Table S2) and sonicated (Branson-Sonifier 250 R, duty cycle 50%, intensity 50%, 5 × 20 pulses, 40 s rest) on ice. The cell lysate was clarified by centrifugation (3-30K Sigma, rotor 12158-H, 14811 x *g*, 4 °C, 45 min). The supernatant was applied to the respective affinity column equilibrated in lysis buffer. For His-tag purification an imidazole containing buffer was used to elute bound proteins. For the purification of MBP-tagged proteins an MBPTrap HP column (5 mL column volume, GE Healthcare) was used with a maltose containing buffer for elution. The Ni^{2+}-IMAC purified protein was further desalted to remove the imidazole. Prior to desalting, the proteins were concentrated (Macrosep Advance Centrifugal Devices, 10 kDa or 30 kDa cut off, Pall) then applied to a PD-10 desalting column (GE Healthcare) and eluted with lysis buffer. Protein concentration was finally determined with a NanoDrop 2000 Spectophotometer (Thermo Scientific). The extinction coefficient as well as the expected molecular weight was calculated with ExPasy ProtParam (Table S3). The purified proteins were stored on ice for not longer than 12 hours until they were used for bioenzymatic reactions.

Table S2. Buffers used for protein purification.

Buffer	Composition
IMAC Lysis-buffer	50 mM Tris<br>100 mM NaCl<br>10% glycerol<br>pH set to 8.0 with HCl
IMAC Wash-buffer	50 mM Tris<br>100 mM NaCl<br>10% glycerol<br>10 mM imidazole<br>pH set to 8.0 with HCl
IMAC-Elution buffer	50 mM Tris<br>100 mM NaCl<br>250 mM imidazole<br>10% glycerol<br>pH set to 8.0 with HCl
MBP Lysis-buffer	20 mM HEPES<br>100 mM NaCl<br>2 mM EDTA<br>5 mM DTT
MBP Elution buffer	20 mM Tris<br>20 mM NaCl<br>1 mM EDTA<br>10 mM maltose

Table S3. Molecular weight and extinction coefficient as calculated with ExPasy ProtParam.

	Molecular weight [kDa]	Extinction coefficient [$M^{-1}cm^{-1}$]
*Ec*PPK1 (P0A7B1)	106.76	115 19
*Vc*PPK1 (A0A0E4CID8)	108.10	100 87
*Sm*PPK2-I (Q92SA6)	36.8	57 41
*Cg*PPK2-I (Q8NM65)	38.21	65 89

UV-Vis analysis:

Concentrations of stock solutions (AMP, ADP, ATP) were determined by UV-Vis spectroscopy using a Jasco V-730 spectrophotometer supplemented with a PSC-736 cell holder. Measurements were conducted in 1 mL quartz cuvettes (QS 1 000). For the measurement, 200 µL KP_i buffer (500 mM, pH 7.5) was mixed with 790 µL of ddH2O and 10 µL of the analyte solution. If the absorption was higher than 1.0, the stock solution was diluted until absorption was below 1.0. Samples were prepared in triplicates and measured three times with Spectra Manager (Version 2.12.00). The exact concentration of the stock solution was determined according to Lambert-Beer using the λ = 259 nm absorption maximum of adenosine nucleotides. The specific absorption coefficient ε = 15400 [$M^{-1}cm^{-1}$] was used for all the three phosphorylation stages [2].

Biocatalytic reactions:

The enzymatic reaction mixture (1 mL) contained final concentrations of 50 mM Tris (pH adjusted to 8.0 with HCl), 20 mM $MgCl_2$, 20 mM polyP (calculated as single phosphates) and 2 mM (or 0.5 or 4 mM) of either ADP or ATP. The respective enzyme was added in a final concentration of 1 µM. The reactions were incubated at 37 °C while shaking at 300 rpm in a thermal shaker. Samples were withdrawn after various time points and frozen in liquid nitrogen. Prior to analysis the samples were thawed at 4 °C, then the protein was removed by passing through a centrifugal filter (Vivaspin 500, 10,000 MWCO Pes, Sartorius) before being analysed by HPLC. All reactions were carried out in triplicates.

HPLC analysis:

HPLC analysis was carried out on an Agilent 12600 Infinity II system using an ISAsphere 100-5 C18 column (250 mm, 4 mm, 5 µm). The gradient uses a sodium acetate buffer A (50 mm, titrated to pH 4.2 with acetic acid) and acetonitrile B (Table S4). For analysis the area under curve was used as calculated by the OpenLAB CDS (Rev. C.01.07 SR3) user software.

Table S4. General HPLC parameters and gradient program for nucleotide separation.

Detection wavelength	254 nm
Flow rate	0.5 mL/min
Sample injection volume	10 µL
Gradient	10 min 98% A
	6 min 98% →70% A
	2 min 70% A
	2 min 70% →98% A
	10 min 98% A

Table S5. Equations obtained for calibration curves of AMP, ADP and ATP.

Substance	y=m*a±b	R^2
AMP	y=13405.2±598.6	0.9940
ADP	y=12057.5±1163.1	0.9726
ATP	y=12837.6±404.5	0.9970

NMR spectroscopy:

NMR spectra of polyP were recorded on a Bruker Avance Neo 400 MHz (162 MHz for ^{31}P) with broadband cryoprobe Prodigy. ^{31}P-NMR spectra were measured using ^{1}H-broad band decoupling. Samples were prepared on ice by diluting the polyP stock solution (1 M) with *dd*H_2O, then the samples were kept at 4 °C to prevent possible hydrolysis. The evaluation of NMR-spectra was done using the software MestreNova from Mestrelab Research.

ePC-SAFT modelling:

The ePC-SAFT predictions used model parameters from the literature, and no additional parameters were used [3,4]. These parameters include the pure-component parameters for ATP, ADP and water and the binary parameters between ATP/water and ADP/water in the framework ePC-SAFT revised model of 2014 [5]. Such parameters were also used in most recent works to explain the thermodynamic feasibility of glycolysis [6]. Please note, that ATP, ADP and water were considered as uncharged molecules, while in the explorative study of reaction mixtures containing orthophosphate also charges were explicitly considered. ePC-SAFT calculates the residual Helmholtz energy a^{res} of any mixture as sum of energy contributions caused by repulsion of hard chains (a^{hc}), attraction of the uncharged chains due to van der Waals dispersion forces (a^{disp}) and hydrogen bonds (a^{assoc}), while charge-charge interactions are also accounted for by the Debye-Hückel theory (a^{DH}). Based on a^{res}, fugacity coefficients are accessible by derivation of a^{res} with respect to system density as well as the mole fraction of the considered species. This can be found in the original ePC-SAFT revised publication [5]. Finally, activity coefficients which are required for the activity-coefficient ratio in multi-component mixtures were predicted by the ratio of fugacity coefficient of a species at the respective finite equilibrium concentration (e.g., 0.5 mM or 2 mM) with respect to the fugacity coefficient of the same species at infinite dilution in water

Supplementary data:

Table S6. Kinetic parameters of selected PPK1 and PPK2 enzymes. Their kinetics for the two different reactions are given.

Enzyme	$K_{M\,(NTP)}$ [µM] ATP synthesis<br>$K_{M\,(NTP)}$ [µM] polyP synthesis	v_{max} [pmol·min^{-1}·mg^{-1}] ATP synthesis<br>v_{max} [pmol·min^{-1}·mg^{-1}] polyP synthesis
*Vc*PPK1 [7]	0.12	$95 \cdot 10^6$
	0.05	$1.6 \cdot 10^6$
*Pa*PPK2 [8]	0.75	$4.6 \cdot 10^8$
	0.5	$7.6 \cdot 10^6$
*Sm*PPK2 [9]	0.032	$2.3–13.7 \cdot 10^6$
	not observed	not observed
*Ec*PPK1 [10]	0.25	$3.7 \cdot 10^6$
	2	$51 \cdot 10^6$
*Pa*PPK1 [8]	not given/ determined	$5.1 \cdot 10^5$
	not given/ determined	$2.1 \cdot 10^6$
*Cg*PPK2 [11]	0.04	$4 \cdot 10^6$
	0.17	$31 \cdot 10^6$

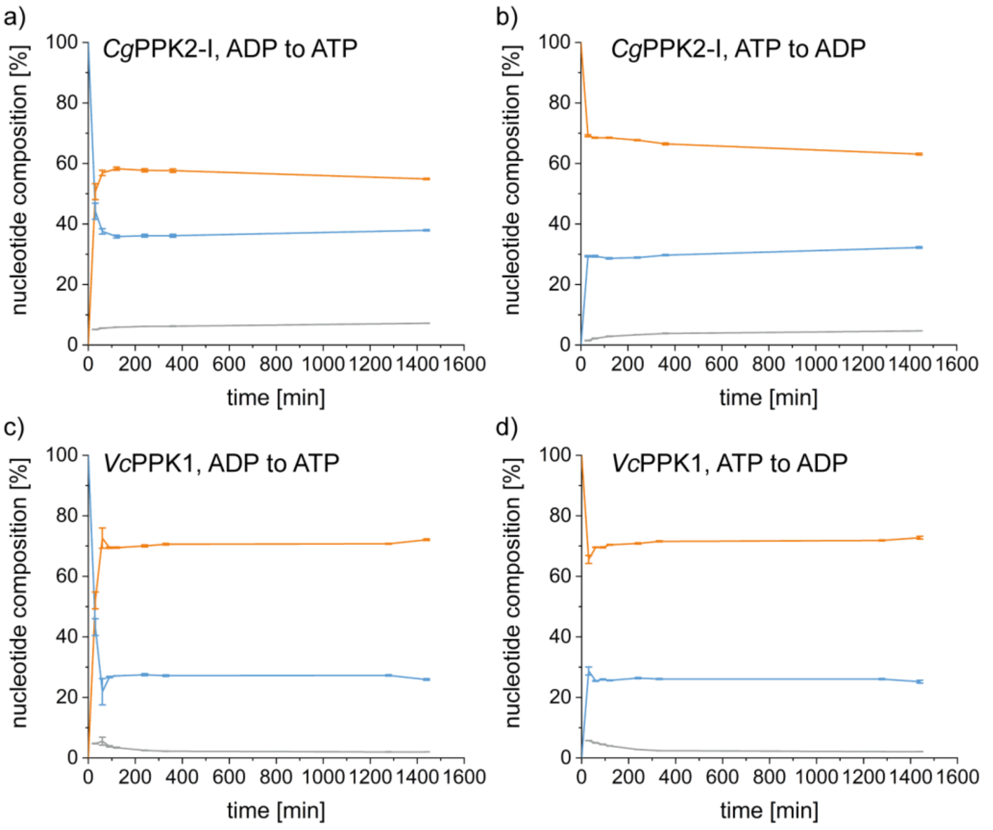

Figure S1. Time courses with 2.0 mM nucleotide.

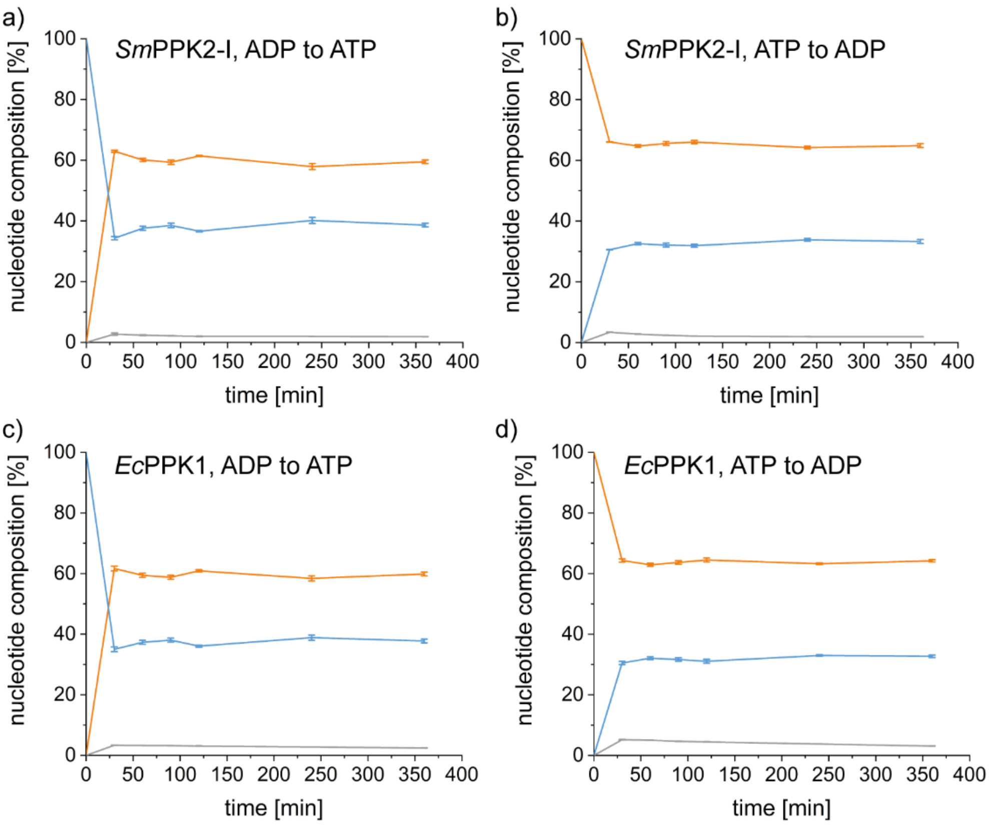

Figure S2. Time courses with 0.5 mM nucleotide.

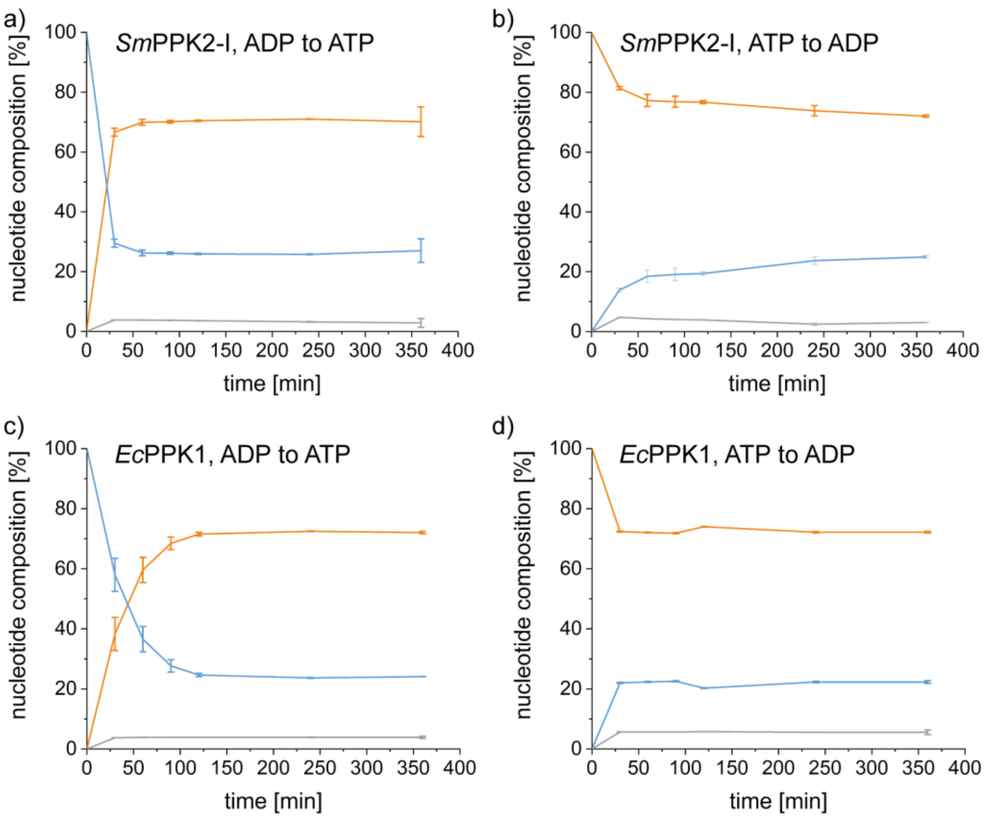

Figure S3. Time courses with 4.0 mM nucleotide.

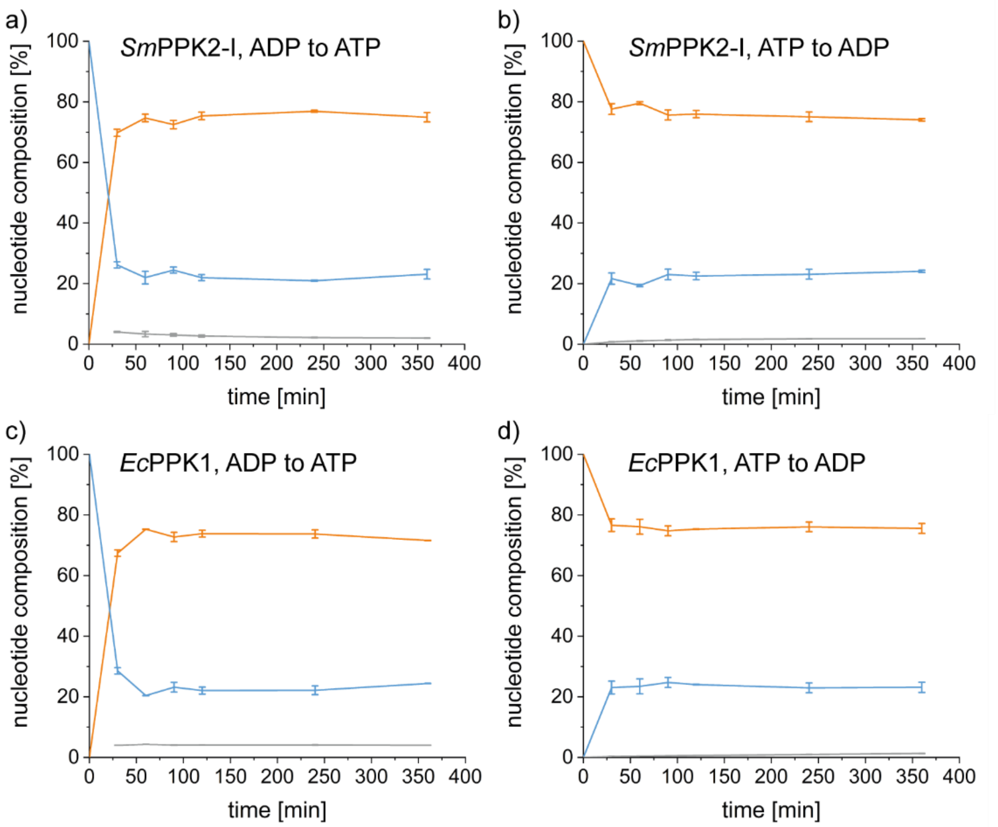

Figure S4. Time courses with 2.0 mM nucleotide and longer chain $polyP_{100}$.

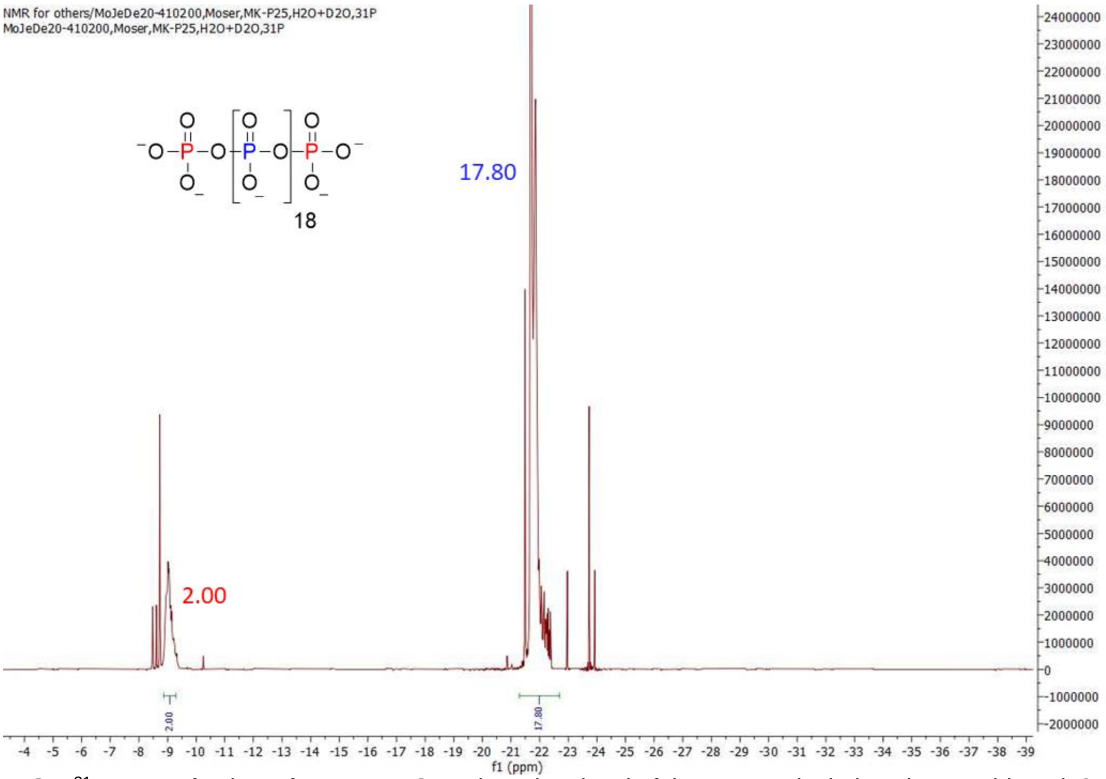

Figure S5. ^{31}P-NMR of polyP$_{20}$ from Acros Organics. The signal of the two terminal phosphate residues (−9 ppm) is distinct from the internal phosphates (−22 ppm). Specifying the integral of the terminal signal allows to conclude the number of internal phosphates by the integral of this signal, in the case of the short chain polyP, the internal phosphates integral was 18, thus the whole chain is in average made of 20 phosphate residues.

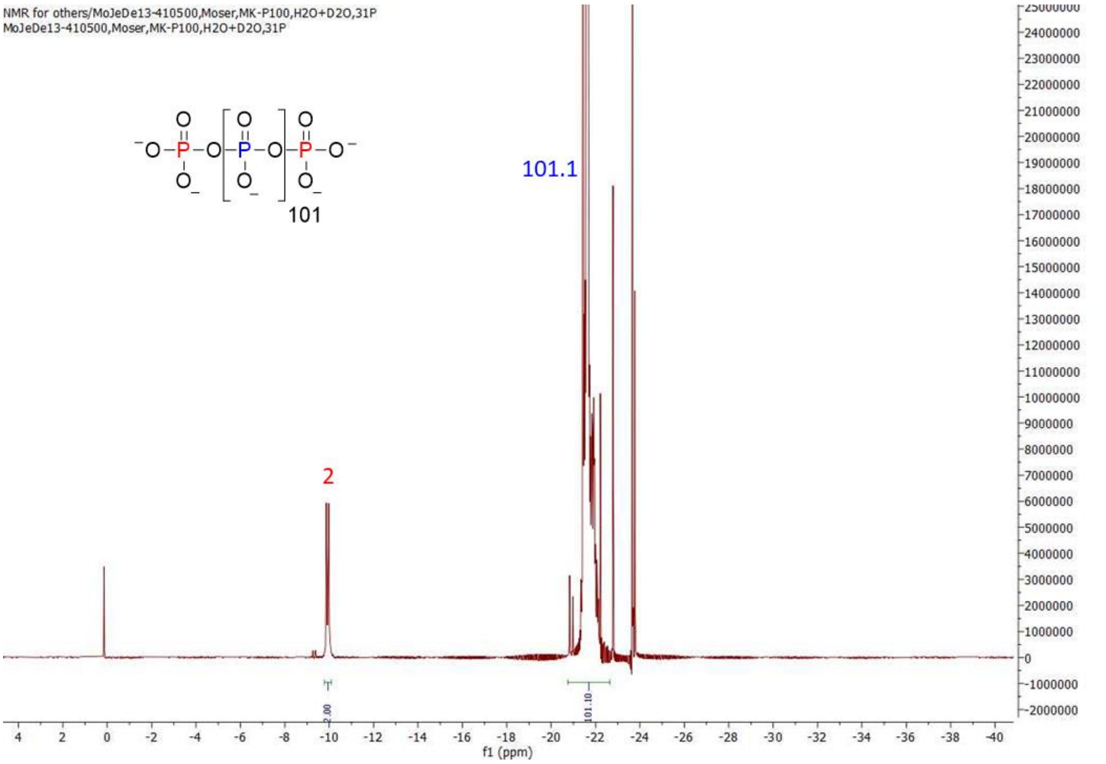

Figure S6. ^{31}P-NMR of polyP$_{103}$ from Kerafast. The same procedure as described in Fig. S5 was applied, resulting in a polyP of average chain length of 103 phosphates.

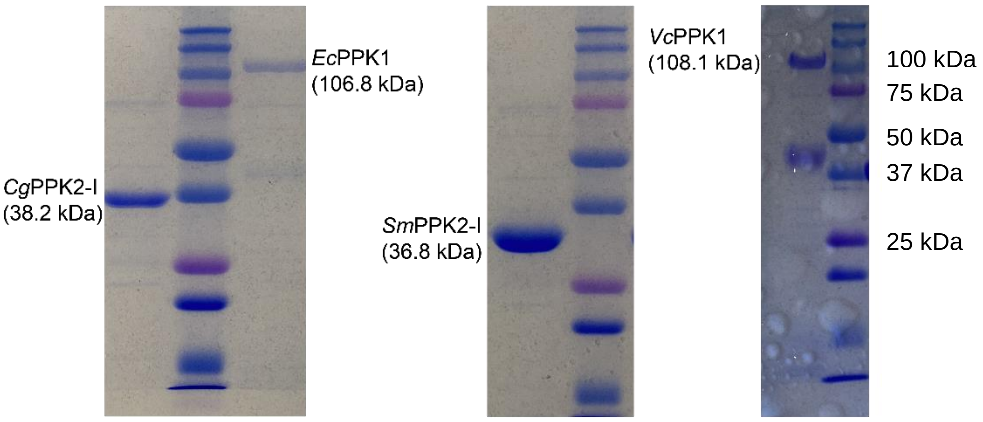

Figure S7. SDS-PAGE of purified enzymes. The Precision Plus Protein Dual Color Standards (Bio-Rad Laboratories GmbH, Feldkirchen, Germany) was used as size reference. For VcPPK1 the enzyme preparation contains some impurity, from the apparent molecular weight we assume that the preparation contained some unspecifically cleaved MBP-tag (42 kDa) which matches the size of the impurity.

Supplemental references:

(1) Mordhorst, S.; Siegrist, J.; Müller, M.; Richter, M.; Andexer, J. N. *Angew. Chem. Int. Ed.* **2017**, *56*, 4037–4041.

(2) Gerstein, A. S. *Molecular Biology Problem Solver: A Laboratory Guide* **2001**, *267.*

(3) Meurer, F.; Do, H. T.; Sadowski, G.; Held, C. *Biophys. Chem.* **2017**, *223*, 30–38.

(4) Meurer, F.; Bobrownik, M.; Sadowski, G.; Held, C. *Biochemistry* **2016**, *55*, 5665–5674.

(5) Held, C.; Reschke, T.; Mohammad, S.; Luza, A.; Sadowski, G. *Chem. Eng. Res. Des.* **2014**, *92*, 2884–2897.

(6) Greinert, T.; Vogel, K.; Maskow, T.; Held, C. *Sci. Rep.* **2021**, *11*, 1–9.

(7) Ogawa, N.; Tzeng, C.-M.; Fraley, C. D.; Kornberg, A. *J. Bacteriol. Res.* **2000**, *182*, 6687–6693.

(8) Zhang, H.; Ishige, K.; Kornberg, A. *Proc. Natl. Acad. Sci. U. S. A.* **2002**, *99*, 16678–16683.

(9) Nocek, B.; Kochinyan, S.; Proudfoot, M.; Brown, G.; Evdokimova, E.; Osipiuk, J.; Edwards, A. M.; Savchenko, A.; Joachimiak, A.; Yakunin, A. F. *Proc. Natl. Acad. Sci. U. S. A.* **2008**, *105*, 17730–17735.

(10) Ahn, K.; Kornberg, A. *J. Biol. Chem.* **1990**, *265*, 11734–11739.

(11) Lindner, S. N.; Vidaurre, D.; Willbold, S.; Schoberth, S. M.; Wendisch, V. F. *Appl. Environ. Microbiol.* **2007**, *73*, 5026–5033.
